# Aberrant Type I Interferon Regulation in Autoimmunity: Opposite Directions in MS and SLE, Shaped by Evolution and Body Ecology

**DOI:** 10.3389/fimmu.2013.00281

**Published:** 2013-09-17

**Authors:** Anthony T. Reder, Xuan Feng

**Affiliations:** ^1^Department of Neurology, The University of Chicago, Chicago, IL, USA

**Keywords:** interferon-beta, Devic’s disease, multiple sclerosis, neuromyelitis optica, phospho-serine-STAT1, SLE, statins, Trex1

## Abstract

Studying the action of mechanisms of type I interferon (IFN) provides the insight to elucidate the cause and therapy for autoimmune diseases. There are high IFN responses in some diseases such as connective tissue diseases, but low responses in multiple sclerosis. Distinct IFN features lead us to understand pathology of a spectrum of autoimmune diseases and help us to search genetic changes, gene expression, and biomarkers for diagnosis, disease progression, and treatment response.

## Prologue

### Evolution of type I interferons: Implications for autoimmunity and cell protection

Interferons (IFNs) were present in early bony fish during the Devonian Period, 400,000,000 years ago. Fish have branched into more species than all other vertebrates combined, and changes in their proteins evolved in parallel with taxonomic diversity. Sequence and structural similarities in fish IFNs suggest that there was an earlier ancestor in common with mammals. Present-day fish IFN has introns and in that way resembles human IFN-lambda, a type III IFN. Mammalian type I IFN has no introns, and may have arisen from retrotransposition of spliced RNA to intronless DNA with the same sequence as the parent gene (1). DNA without introns is more easily duplicated, leading to many subtypes. Transcription from DNA without introns is rapid and can bypass mechanisms that can be derailed by virus infections. Anti-viral IFN genes would therefore have a selection advantage during fevers.

Interferons vary widely between species of present-day fish. Salmon have 11 subtypes of type I and III IFN; zebrafish have two group 1 and two group 2 subtypes. Subtypes of IFN likely evolved to effectively target different viruses in vertebrates. Viruses bind toll-like receptors (TLRs) 3, 7, 8, and 9 and retinoic acid-inducible gene-I (RIG-I) receptors. These fish receptors then induce IFNs which activate or induce type I IFN receptors (IFNARs), and in turn, signaling proteins (JAK1, TYK2), transcription factors (STAT1), and many IFN-stimulated gene (ISG) proteins (such as IRF, MxA, PKR, and viperin) (2). All are present in humans.

The human type I IFN family comprises 13 IFN-α, 1 IFN-β, 1 IFN-κ (keratinocytes), 1 IFN-ω (Table 1). IFN-τ is a related variant found only in the ruminant trophectoderm, important early in pregnancy. IFN-δ is produced by pig trophoblasts. IFN-ζ/limitin is present in mice.

**Table 1:** **Interferons in mice vs. humans**.

	Mouse	Man
**Type I IFNs**	14 IFN-α genes	13 IFN-α genes (12 proteins)
19 kDa, 165–166 aa	Four have alleles that differ between strains	
	10 Glycosylated	2 Glycosylated
	IFN-α4 promoter binds IRF3	IFN-α4 promoter binds IRF3
20 kDa, 22 kDa glycosylated, 166 aa	1 IFN-β	1 IFN-β
	IFN-β promoter binds IRF3	IFN-β promoter binds IRF3
22 kDa and glycosylated, 187 aa	1 IFN-ε	1 IFN-ε
25 kDa, 180 aa	1 IFN-κ	1 IFN-κ
20 kDa, 172 aa	1 IFN-ω	None
19 kDa, 182 aa	1 IFN-ζ (limitin)	1 IFN-ζ (limitin)
**Type II IFN**	1 IFN-γ	1 IFN-γ
17 kDa		
**Type III IFN**		
21 kDa, 26–35 glycosylated	None	IFN-l1 (IL-29)
22 kDa, 24 glycosylated	IFN-l2 (IL-28A)	IFN-l2 (IL-28A)
21 kDa, 24 glycosylated	IFN-l3 (IL-28B)	IFN-l3 (IL-28B)

**Signaling**	**P-STAT proteins**	**P-STAT proteins**

**STAT1a** 91 kDa	P-Y701-STAT1	P-Y701-STAT1
	P-S727-STAT1	P-S727-STAT1
**STAT1b** 84 kDa	P-Y701-STAT1	P-Y701-STAT1
**STAT2** 113 kDa	P-Y689-STAT2	P-Y690-STAT2
	No Y833 – truncated (76)*	P-Y833-STAT2
	No Y841 – truncated	P-Y841-STAT2
	No STAT2 induction of P-STAT4 (76)	P-STAT2 induces P-STAT4
**STAT4** 86 kDa	Type I IFNs induce Th1	P-STAT4 induces IFN-g, Th1
		Type I IFNs induce Th1, but reports in MS are mixed

**EAE vs. MS**	**EAE**	**MS**

**The target**	Antigen-induced	No known antigen (ADEM is Ag-induced)
**Therapy**	Improvement with pure IFN-β	Most improve with IFN-β therapy (∼85% have a low IFN signature) (∼15% have higher IFN signature; less response to IFN-β therapy)

*“Humans do not provide a good model for mouse immunology” from Ref. (76).

Humans have three type III IFN-λs (*aka* IL-29, IL-28A, and IL-28B) which bind a different receptor (IL-10Rβ and IL-28Rα chains), often on epithelial cells and liver cells. IFN-lambda has introns. Only restricted cell types express the unique type III IFNAR, and thus it may have fewer side effects. It is produced by plasmacytoid dendritic cells (pDCs) and induces IL-6, IL-8, and IL-10, and activates type II monocytes. It is anti-proliferative, but only for several tumor cell subtypes.

Viruses were likely to have been early targets of IFNs in evolutionary history. There is intense selective pressure to rapidly destroy viruses and virus-infected cells. Human interferons induce proinflammatory cytokines and chemokines such as CXCL10 (IP10), and turn on hundreds of anti-viral genes such as MxA, viperin, 2′,5′-OAS, and PKR. IFNs also activate cytolytic T cells, NK cells, Th1 cells, macrophages, and cells from other tissues, and induce apoptosis of infected cells. (Control of inflammation is discussed below.)

Interferons regulate a 1000 genes (3, 4). Many of these genes are not anti-viral. Some IFN-regulated genes shape the innate and adaptive immune systems, some modify transcription, some are anti-proliferative and pro-apoptotic to combat cancer, other genes control fertility, fatty acid oxidation, free radical neutralization, energy metabolism, and cell protection and tissue repair. These functions are a leap beyond the anti-viral role of IFNs. Virus-independent benefits suggest that IFN responses evolved to “clean up the mess” after a virus infection (Ed Croze, personal communication, 2007). Subnormal IFN levels, as in multiple sclerosis (MS), could disturb immune regulation and also diminish cell protection. Therapy of MS with type I IFNs reverses these disturbances.

## IFN Signaling, Kinetics, and Species Differences

The primary source of IFN is from pDCs (IFN-α > IFN-β production), fibroblasts – the major producers of IFN-β (IFN-β > IFN-α), macrophages, and endothelial cells. pDC are only 1% of DC. Myeloid dendritic cells (mDCs) secrete IL-12 and other cytokines, but only small amounts of IFN.

Interferons signal through a rapid cascade. Type I IFN is secreted within an hour of stimulation with virus or poly(IC), a synthetic analog of viral double-stranded RNA (dsRNA). Viral nucleic acids are recognized by pattern receptors (described below) that activate IRF3, which then turns on IFN-β and IFN-α1 synthesis. These first subtypes of IFN bind IFNARs and activate IRF7 in surrounding cells to induce multiple other type I IFN subtypes/species.

The cell surface type I IFNAR has two-chains, IFNAR1 and IFNAR2 which activate TYK2 and JAK1. Phospho-JAK1 and TYK2 then phosphorylate STAT1 (P-Tyr701-STAT1) and STAT2 (P-Tyr-STAT2) proteins which complex with IRF9 (p48) (ICSBP) to form IFN-stimulated gene factor 3 (ISGF3). Like type I IFNs, IFN-lambda induces ISGF3. Within 10 min after IFN activates its receptor, the ISGF3 binds the IFN-stimulated response element (ISRE) of a large group of gene promoters. DNA-bound P-Y-STAT1 is sometimes then phosphorylated on serine 727 within ∼10 min (forming P-S-STAT1) (5) which boosts the signal for a subset of IFN-regulated genes. STAT2 has no serine phosphorylation site.

## Type I IFN Regulation and Localization of IFN Production

Once the receptors above are activated, a sequence of intracellular signals amplifies IFN secretion. IRF3 is phosphorylated, binds to DNA promoters, and then IFN-β and IFN-α1 (IFN-α4 in mice) are rapidly secreted. These two IFNs then bind the type I IFNAR on the same and other cells and activate IRF7 which binds to promoters for other IFN-α subtypes.

Other intracellular signal transduction molecules modify the JAK-STAT pathway, including, PI3K, CRKL, RAP1, PKC-δ and ε, and p38 in the MAPK cascade (6). These converging proteins are cell-specific. These pathways enhance effects of type I IFNs, but perhaps the most important interaction is with IFN-γ.

Type II IFN-γ is the prototypic immunoregulatory Th1 cytokine, but it is only distantly related to type I IFNs and has only weak anti-viral effects. It activates STAT1 on tyrosine. P-Y-STAT1 homodimers bind to the gamma-activated site (GAS), present in a set of promoters that are different from the type I IFN ISRE that is activated by the STAT1/STAT2/IRF9 complex. IFN-γ-activated P-Y-STAT1 can interact with IFN-β-induced P-Y-STAT1. Preincubation with IFN-γ “primes” cells for a more vigorous response to IFN-β.

Interferon signaling differs between mice and men. In humans, activation of STAT2 then phosphorylates STAT4, which can turn on more cytokine genes. In some mouse strains, however, STAT2 is truncated, and does not activate STAT4 (Table 1). Thus, the murine experimental allergic encephalomyelitis (EAE) model of MS suffers from being an antigen-specific model of a disease with no known antigen, and because inflammation in EAE is regulated by interferon signaling that is missing part of the human signaling cascade.

Some downstream genes are rapidly induced (e.g., anti-viral and immunoregulatory genes); others take longer to plateau (e.g., genes with secondary induction, e.g., dual oxidase 2) (4). Kinetics also vary between cells. For instance, IL-10 mRNA production by activated monocytes is maximal at 4–8 h and is inhibited by IFN-β. In activated T cells, the peak is at 24 h and IL-10 mRNA is amplified by IFN-β (7).

Interferon-β, compared to the individual subtypes of IFN-α, induces a larger number of genes in human fibrosarcoma cells, and does so more quickly (3). IFN-β binds to the IFNAR for a longer time than IFN-α (8), explaining the differential gene induction. Perhaps because of the greater number, but more balanced portfolio, of induced genes, IFN-β has fewer side effects than equivalent anti-viral doses of IFN-α. IFN-β is also more effective than IFN-α in some therapies. Despite the traditional use of IFN-α subtype to treat some forms of cancer, IFN-β is actually more potent against several types of cancer at equivalent anti-viral titers of both IFNs (9). It is also more effective in MS therapy. (Cytoprotective effects are discussed with MS, below.)

Does IFN-β cross the blood-brain barrier (BBB)? Although CSF IFN levels are only 1/1000 of serum levels in a normal monkey (10), the damaged BBB in MS and EAE could allow IFN to cross. In humans, circumstantial evidence suggests that IFN-β has direct effect on the CNS. IL-10 levels in CSF rise after IFN-β-1a therapy (11) and black hole formation is reduced by IFN-β-1b (12). In mice with EAE, IFN-induced mRNA is clearly present in the CNS, after controlling for the effects of EAE and IFN-induced mRNA outside the CNS (13). Thus, IFN-β may have direct effects on brain cells in MS.

## Triggers for Type I IFN Production

Virus components that induce type I IFNs include exogenous virus RNA or DNA and associated proteins, vaccines which are attenuated viruses or contain virus components, and endogenous retroviruses that make up ∼8% of human DNA. Exuberant responses to viruses or to abnormally processed DNA from dying cells and their nucleic acids can activate the immune system and lead to autoimmune diseases such as systemic lupus erythematosus (SLE).

Receptor families recognize conserved pathogen-associated molecular patterns (PAMPs; i.e., “danger” from viral nucleic acids and other motifs) and viruses. These receptors include TLR, RIG-I, melanoma differentiation-associated protein 5 (MDA5), CD11b/CD18 (Mac1), STING (DNase II), as well as the Trex system. Each virus can be detected by multiple PAMP receptors.

Toll-like receptors were originally characterized in fruit flies, where they recognize a developmental growth factor and control antimicrobial responses in adult flies. In humans, 10 types of TLR recognize bacterial components, RNA, and DNA. Fibroblasts express TLR3 on their surface. Other, intracellular, sensors detect viruses after they are internalized or generated inside cells. They also sense abnormally processed nucleic acids in connective tissue diseases after Fc receptors internalize DNA-Ab complexes. TLR3 is endosomal in monocytes and mDCs (TLR3 is not in pDC). Virus RNA is recognized after phagocytosis and internalization, or after enveloped viruses penetrate the cell by endocytosis. After binding dsRNA or pIC, human TLR3 is activated within acidified cytoplasmic endosomes (14). TLR3 activates TRIF, and then kinases (IKKε, TBK1) that phosphorylate IRF3 and IRF7 (below). TLR7 and TLR8 bind single-stranded RNA (ssRNA), poly-IC, and imiquimod. TLR9 binds intracytoplasmic viral or bacterial CpG-rich DNA. TLR7, 8, and 9 are present in pDC.

The RNA helicases, RIG-I and MDA5, bind short and long viral dsRNA, or pIC. Homologs of all of these receptors, with the exception of the OAS system, are present in fish where they sense pathogens and induce IFNs and many ISG.

IRF3 is constitutively expressed at high levels in most cells. IRF7 is present at only low levels, mainly in immune cells especially pDC, but is necessary for the initial induction of IFNs. Activated IRF3 induces IFN-β and IFN-α1 (IFN-α4 in mice) which prime the type I IFN system for a much stronger response. After virus exposure, rapidly secreted IFN-β binds the IFNAR and induces and activates intracellular IRF7 (at 4 h), while IRF3 is degraded. IRF7 broadens the response by inducing multiple IFN-α genes in pDC which and activate pDC, T cytolytic, Th1, and NK cells. In parallel, virus-exposed conventional DC and monocytes secrete cytokines such as IL-12 plus low amounts of IFN-α1 and IFN-β.

TLR7, 8, and 9 are expressed at high levels in pDC, and activate MyD88. These TLRs activate IRF7 which induces transcription of some IFN-β plus high levels of multiple types of IFN-α in pDC (15). Ligation of RIG-I and MDA5 activate transcription factors IRF3 and NF-κB, which travel to the nucleus and initiate transcription of type I IFNs.

## Consequences of Excess Stimulation by DNA, RNA, Bacteria, and Cytokines

Influenza infections elevate type I IFNs in serum and pulmonary secretions. In Sjögren’s syndrome, foci of inflammation in salivary glands are positive for IFN-α, and serum IFN levels are elevated. In SLE and Sjögren’s disease, high interferon levels and lack of immune regulation cause damage to target organs. Surprisingly, because it is an immunologically privileged site, the brain also exhibits IFN expression or binding. An early paper showed IFN-α-positive macrophages, IFN-γ-positive astrocytes and microglia, and occasional IFN-β-positive astrocytes and macrophages, within active chronic MS brain lesions (16). During chronic hepatitis C therapy, IFN-α (5 MU TIW) reduces PET activity by 10% in the pre-frontal cortex (17), and 3 MU TIW causes gradual slowing of VEP over 1 year (18). Hepatitis C in these patients may have had additive effects with IFN-α. In contrast, VEP slowing is likely not seen with IFN-β therapy, as P300 potentials are stable or improved (19). Excessive CNS IFN levels cause encephalopathy in Aicardi–Goutières syndrome, Cree encephalitis, and cerebral malaria, *v.i*. (20), and possibly in SLE and Sjögren’s disease.

The Trex system is an intracellular monitor for products of endogenous retroviruses. Trex1 (DNAse III) is the major 3′,5′-exonuclease in humans. The single-exon gene codes for a cytosolic protein that is induced by the IRF3-dependent IFN-stimulated response to foreign DNA (21). It edits DNA by stripping off one stand of ssDNA and metabolizes intranuclear DNA; residual ssDNA that escapes from the nucleus can trigger an immune response. Remnants of ancient retroviruses human endogenous retrovirus (hERV) that have incorporated into the DNA comprise ∼8% of the human genome, and retroelements may outnumber our genes by 100-fold (21). These hERV are not complete virions, but portions can be transcribed, and do generate ssDNA fragments and proteins. hERV DNA is degraded by Trex1. In Trex1 knockout mice, 22% of the DNA in inflamed myocardium is coded by endogenous retroviruses, vs. 7% in wild type mice (21), indicating that Trex1 is needed to destroy these retroviral genes. Reverse-transcribed DNA that induces IFN production is the principle cause of autoimmunity in these mice. Defects in Trex1 lead to high circulating levels of foreign DNA, which triggers type I IFN production and autoantibody production.

Trex1 deficiency causes constitutive activation of the systems that control ATM-dependent double-strand breaks and cell cycle checkpoints. Induction of ATM for DNA-damage monitoring also activates p53 and BRCA1, leading to fewer tumors, self-renewal of hematopoietic stem cells, thymocyte survival, but less apoptosis of autoreactive immune cells. This could amplify autoimmune disease.

A defective Trex1 gene is common in Canadian Cree Indians, and causes excessive interferon production. This leads to Cree Indian Encephalopathy, or Aicardi–Goutières syndrome, an encephalopathy associated with lupus-like symptoms (22). Excessive CSF IFN-α (42 IU/ml) in affected children (Pierre Lebon, Paris) mimicked congenital viral infection, but associated chilblains (pernio) pointed to SLE and autoimmunity, and IFN-α. These children have: (1) progressive microcephaly and failure to attain neurodevelopmental milestones, beginning in early infancy; (2) recurrent viral, bacterial, and fungal infections; (3) cerebral atrophy, white matter attenuation, and calcifications of basal ganglia, white matter, and/or cerebellum on CT scan; (4) perivascular chronic inflammatory infiltrates in cerebral hemisphere white matter and hyperplasia of vascular endothelial linings; and (5) polyclonal hypergammaglobulinemia. They sometimes have corroborative features such as: (1) dystonic posture; (2) systemic autoimmune abnormalities – high ALT and Abs to cardiolipin, ssDNA, dsDNA, and RNA-protein complexes; (3) splenomegaly and lymphadenopathy; (4) CSF pleocytosis with high CSF IFN-α and Ig; high blood CD8 “suppressor” and B cells; (5) intermittent hyperpyrexia; (6) chronic active Epstein–Barr virus (EBV) or CMV infection or persistent viral excretion; (7) similarly affected siblings; and (8) acrocyanosis with autoamputation (23, 24). With this “chilblain lupus” from Trex1 deficiency, cold exposure causes cyanosis of toes and fingers because of damage to capillaries. Eighty percent of families with Aicardi–Goutières syndrome have mutations in one of four nuclease genes – the exonuclease Trex1 [chromosome 3p21 (*AGS1*)] or the genes for all three components of the ribonuclease H2 enzyme complex (*AGS2, 3*, and *4*).

In SLE, apoptotic products of PMN, T cells, and macrophages are not cleared correctly by macrophages. Apoptotic blebs contain modified chromatin, and neutrophil extracellular traps are released by dying neutrophils (NETosis). Abnormally processed nucleic acid-containing debris circulates as phospho-DNA that is recognized as a virus, or Ab-DNA neoantigens, that activate FcR and TLR of DC and the BCR of B cells. Chromatin, double-stranded DNA, and RNA-binding ribonucleoproteins activate an autoimmune circuit and production of IFN-α/β by pDCs and anti-dsDNA Abs by autoreactive B cells.

Excess local IFN-α damages the CNS. Encephalopathy develops when IFN-α is overexpressed in astrocytes (25). Transgenic mice develop early mineralization around blood vessels in the thalamus at 2 months, calcium crystals in cerebellum at 12 months, and perivascular CD4+ T cell infiltrates in the CNS. Some pediatric infections lead to high CSF IFN-α (“TORCH,” from Toxoplasmosis, Other, Rubella, CMV, HSV), with sequelae of CNS calcifications and brain atrophy. Chronic exposure to IFN-α in cultured astrocytes increases GFAP expression, reduces proliferation, and causes hypertrophy and activation (25, 26), reflecting the changes from high CNS IFN-α in Aicardi–Goutières syndrome.

In MS, HERV DNA and antibodies to HERV proteins appear in serum, CSF, and brain (27). Activated immune cells release HERV nucleic acids into the cytosol. This could induce type I IFNs in immune cells or the CNS in some MS patients.

“Interferon inducers” also generate non-IFN cytokines and proteins. For instance, pIC is a potent stimulus for lymphocyte production of ACTH and other proteins processed from the pro-opiomelanocortin (POMC) precursor molecule (28). IFN-α2 triples serum ACTH and cortisol 5 h after injections in patients with hepatitis B; flu-like symptoms do not correlate with induction (29). In contrast, IFN-β-1b therapy of MS does not elevate cortisol (30).

Environmental agents and drugs can modify IFN effects. Vitamin A activates STAT1 and synergizes with IFN-β (31). Oral vitamin D3 regulates 63 genes, 62 of these were also regulated by IFN-β-1b therapy in early MS (Munger, uncorrected data analysis of BENEFIT study; personal communication, CSMC, 2013). Statins, which lower cholesterol and are anti-inflammatory, surprisingly block IFN signaling. They reduce formation of P-Y-STAT1 and IFN-β-induced MxA production *in vitro* and *in vivo* (32), and allow attacks of MS when added to ongoing IFN-β-1a therapy (33) (below).

The response to exogenous or endogenous triggers has to be tightly controlled, or unchecked immune responses will destroy the host (34). Excessive responses to influenza, as in the 1918 pandemic, lead to death from severe pneumonitis. Weak immune responses, as in never-exposed youths or in octogenarians, do not control the virus. Prior immune education usually allows rapid clearance, manageable immune reaction, and tolerance/regulation that allows the inflammation to subside. Tolerance should be under stronger selective pressure than the actual anti-pathogen response (34, 35). As a virus is cleared, the immune system tempers inflammation with regulatory T cells, inhibitory receptors for immunoglobulin Fc on immune effector cells, apoptosis and autophagy of target cells, anti-inflammatory cytokines, and induction of intracellular suppressors of cytokine signaling (SOCS) proteins. IFNs regulate each of these immune functions, and regulation of each, had to evolve over eons.

Prevention of anti-self responses in an individual are honed over a lifetime by environmental events which generate a complex immune repertoire to combat danger. If the environmental guidance is missing, holes in the repertoire can trigger autoimmunity to self antigens. If certain antigens or levels of interferon are in excess, immune responses could be excessive and can cross react with self, triggering autoimmunity.

## Disease with High Serum Type I IFN Levels and High Responses to IFN: SLE, CNS Sjögren’s Syndrome, NMO, and a Minority of MS Patients

Early reports identified a unique “acid labile” type I IFN in serum of patients with SLE and HIV infection (36). Type I IFN is resistant to pH 2, but IFN-γ is destroyed on exposure to acid. Acid sensitivity may be from have aberrant glycosylation of some subtypes of IFN-α in connective tissue disease and HIV. More recent studies of patients with SLE demonstrate significant increases in serum type I IFN activity (37, 38) and excessive signatures for IFN-induced RNA in white blood cells [in Ref. (37)]. Responses to self DNA or viruses are inappropriately regulated in hereditable complex traits linked to single nucleotide polymorphisms (SNPs) in IFN-regulatory genes (TYK2, IRF5, STAT4, TNFAIP3, and TREX1) and diverse mutations in Trex1 in 0.5–3%. In support, lupus-prone mice that are transgenic for TLR7.1 and have excessive levels of TLR7 have more autoantibodies and early severe lupus. High IFN-α levels correlate with SLE disease activity and severity in some studies (24). Clinically relevant, therapy with type I IFN causes *de novo* SLE or worsening of preexisting disease.

Nearly all parts of the body can be affected in SLE from the anti-nuclear antibodies and symptoms derived from high serum IFN levels. A constellation of damage affects skin, mucocutaneous tissue, joints, kidneys, lungs, heart, and blood vessels – with immune-complex vasculitis and thrombotic occlusions. CNS lesions are rare, but occasionally lupus myelopathy develops with devastating vasculitis, inflammation, swelling, and demyelination over many cord segments. In addition, serum type I IFN levels are elevated. SLE problems that are possibly related to high serum IFN levels include lymphopenia, myalgia and muscle weakness, joint pain with modest infusions, significant constitutional symptoms – headache, body ache, malaise, and fatigue that can herald neuropsychiatric problems (poor memory, mood swings, seizures, and psychosis – without strokes or vasculitis, although there is underlying small vessel vasculopathy in SLE brains). Cognitive problems are often unrelated to SLE exacerbations, suggesting a second mechanism of damage. Perhaps high levels of type I IFN, and abnormally processed nucleic acids induce autoantibodies that are specific to certain regions of the brain and interfere with neuronal function (39) (Table 2). With disruption of the BBB by stress or epinephrine, neurons in the amygdala are damaged by Abs (anti-NR2) to orphan NMDA receptors. With BBB disruption from infections, auto-Abs damage hippocampal neurons and disrupt spatial memory. In both cases the damage is excitotoxic, without inflammation, due to Ab-mediated stimulation of the NMDA receptor.

**Table 2:** **Characteristics of demyelinating disease**.

	MS (40)	SLE	CNS Sjögren’s (58)	NMO (77, 78)
MRI brain lesions	Periventricular, Dawson’s fingers	Gray and white matter lesions	Centrum seniovale	Hypothalamic and periventricular in <10%
	“Random” WM + GM, but predilection for certain areas			
	Small to large lesions	Small WM, rare large vasculitic, and CVA lesions	Small lesions	Medium to large lesions, later in course
MRI cord lesions	<1 Segment	Rare extensive myelopathy	>3 Segments, also smaller lesions (58)	>3 Segments; longitudinally extensive
	Often subpial or acentric		Central cord	Central cord
Relapse rate	q 2 year	∼Once per 5 year, on therapy	Similar to NMO	Frequent (∼2×/year) early in the course for NMO + patients
Progression	PPMS at onset in 10%	Stepwise and gradual multi-organ failure	Progressive sicca symptoms	None
	RRMS becomes SPMS @ 8–15 year	∼Once per 5 year, on therapy		
Pathology CNS	Demyelination > axonal loss	“Vasculopathy” > cognitive changes	Demyelination < axonal damage	Demyelination < axonal damage
	Many lesions will repair	Rare arteriopathy	Vasculopathy	Vasculopathy
	Destruction by CD8 T cells and monocytes	Cells include PMN and Eos	Severe	Severe and destructive
				No repair
Serum marker	No marker	Anti-dsDNA	SSA/SSB 40%	Anti-AQP-4 60–75%
			Anti-AQP-4 50%	
Target Ag	Unknown	Abnormally processed DNA and RNA	Nucleic acids, AQP-5	AQP-4
			Minor salivary gland inflammation in 100%	
Serum type I IFN	Low IFN-α/β	High IFN-α/β	High IFN-α/β	High IFN-α/β
IFN-β Response by MNC	Low	High	High	High
CSF	High IgG	∼Normal	∼Normal	∼Normal
OGCB	90%	10%	∼10%	20%
Triggers for exacerbation	Virus, vaccination for yellow fever	Virus	Virus, possibly	Virus; UTI (AQP-Z) (79)
		Type I IFNs	Possibly type I IFNs	Possibly type I IFNs
	Low vitamin D	Sunlight		
	Smoking: MS onset and exacerbations	Smoking: SLE onset and exacerbations	Possibly smoking	Possibly smoking
Therapy	IFN-β, glatiramer, natalizumab, fingolimod, fumarate, teriflunomide, alemtuzumab, rituximab, laquinimod	Hydroxychloro-quine, steroids, chemotherapy	Rituximab, steroids, chemotherapy	Rituximab, steroids, chemotherapy
	Sunlight; vitamin D potentiates IFN-β-1b			
	GI parasites			
	Gout is rare			
Pregnancy	Benefit, perhaps from estriol	Worse	Unknown	Unknown
Linked diseases	?Thyroid	Connective tissue diseases	Connective tissue diseases	Connective tissue diseases
	?Ulcerative colitis	Aicardi–Goutieres		

AQP, aquaporin; GI, gastrointestinal; GM, gray matter; NMO, neuromyelitis optica; UTI, urinary tract infection; WM, white matter.

Interferon levels are high in serum and salivary glands in Sjögren’s disease, a connective tissue disorder with dry eyes and mouth (sicca) from inflammation of the lacrimal and salivary glands, along with synovitis, vasculitis, and neuropathy. Classic primary Sjögren’s syndrome typically appears in middle-aged women. CNS involvement is usually not part of primary Sjögren’s, but a CNS variant was recently described (Javed, below). Secondary Sjögren’s syndrome coexists with SLE, polyarteritis nodosa, rheumatoid arthritis, scleroderma, polymyositis, and neuromyelitis optica (NMO) (below). Glands are infiltrated by foci of memory CD4 T cells (secreting IL-10 and IFN-γ), macrophages, and mast cells near activated epithelial cells (IL-1β, IL-6, TNF-α). A small number of infiltrating activated B cells produce high levels of immunoglobulin, some directed against rheumatoid factor, SSA/Ro, SSB/La, anti-nuclear antigens, aquaporin 5, and the M3 muscarinic receptor. The surprising overlap between CNS Sjögren’s syndrome and NMO (often considered an “MS variant”) is discussed below.

## Multiple Sclerosis: Epidemiology, Immunology, and Role of IFN

Multiple sclerosis is an inflammatory CNS disease with no clear etiology, pleomorphic clinical and MRI appearance, and huge variation in clinical course, with a transition from bi-yearly relapses and remissions to a progressive form after ∼8–15 years (40). Clinical attacks plus the 10× more frequent subclinical events seen on MRI cause cumulative damage. Attacks last 2–6 weeks, affect any part of the CNS, and often resolve to near-baseline function. MRI lesions enhance with Gd, from leakage through the BBB and perhaps pinocytosis by activated endothelial cells of post-capillary venules. Lesions enhance for a month, then the residual high-water T2 signal fades slowly or becomes a permanent black hole reflecting damage to axons, neurons, and oligodendroglia. Therapy with IFN-β and some other drugs prevent Gd+ lesions but also prevent black hole formation after Gd+ lesions appear. This suggests that MS therapies may have neuroprotective effects.

Multiple sclerosis is more likely to arise when a first degree relative has MS (10- to 20-fold increase), and in smokers, the obese (2×), in those with little exposure to sunlight or with low vitamin D levels (2×), and when first EBV infection is delayed to adolescence. Once MS develops, exacerbations are more frequent in smokers (1.6×), and after infections by many different viruses or after vaccination with certain attenuated live viruses such as yellow fever (41). IFN-β therapy does not prevent virus infections, but it does diminish the residual clinical deficit after a virus-induced exacerbation of MS (42).

Multiple sclerosis usually begins in the reproductive years and is thee times more common in women than men. A subset of patients, perhaps 25%, has a high IFN signature as well as more clinical and MRI attacks before therapy, and these patients often do not respond to IFN-β therapy (43, 44). They have excessive activation of monocytes and mDCs and a high type I IFN-induced gene signature that predicts 70% of non-responders compared to patients with normal IFN signatures. Non/poor-response was defined as one or more attacks of 2 years on therapy (43) or three or more new or worse MRI lesions (44). However, since high disease and MRI activity predicts more future activity, these patients should be considered partial-responders, in the absence of an untreated control group.

Immune studies and SNPs on GWAS implicate many genes involved in immunoregulation or IFN signaling. HLA-DR2 has the strongest GWAS odds ratio at 2.0; others are only 1.1–1.4, but do include IFN-regulated genes such as Tyk2, 2′,5′-oligoadenylate synthase (OAS1), IRF5, MxA, and many IFN-affected immune response genes (40, 45). This suggests that environmental influences and the education of the immune system are critical in the development of MS and its course. A Th1 bias is characteristic of MS. As a consequence, cancers, virus infections, and allergies is less frequent than expected in MS patients (40). A shift to Th2 immunity, seen with glatiramer therapy or after parasite infestation, reduces exacerbations of MS. IFN-β, however, does not simply cause a shift to Th2 immunity – type I IFNs typically enhance Th1 immunity (46). In the CSF of IFN-β treated patients, however, IL-10 is elevated (11), perhaps from IFN-β stimulation of activated T cells (7).

It would seem that MS and connective tissue disease could have a common etiology because they are “autoimmune.” However, MS only rarely coincides with SLE (47), NMO is a separate entity, and the majority of MS patients benefit from IFN-β therapy instead of worsening (48). Importantly, the vigorous signature in SLE, Sjögren’s, and NMO, contrasts with the subnormal serum type I IFN activity and WBC responses to IFN in most MS patients (Table 2).

## Multiple Sclerosis: Low Serum Type I IFN Activity and Weak Responses to IFN

**Figure 1:**
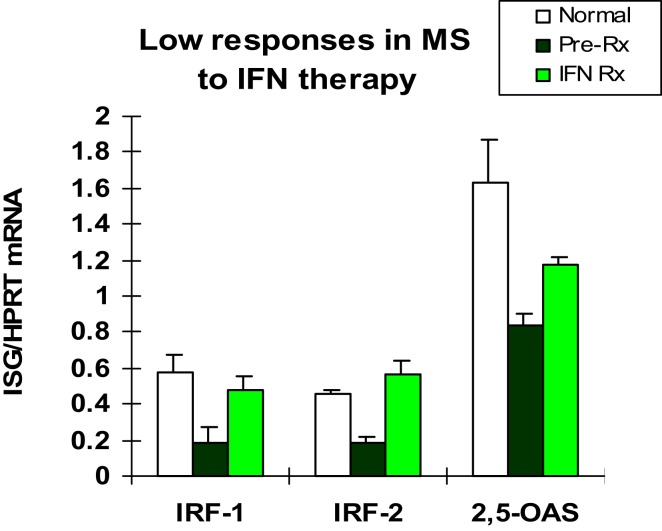
**Basal IFN-regulated mRNA levels in therapy-naïve stable RRMS (black bars) are lower than in healthy controls (white)**. IFN-β therapy induces mRNA production in MS MNC (Pre-Rx vs. IFN Rx), but levels are still at or below levels in *unstimulated* normal MNC (white). MNC assayed before and 3 months after *in vivo* IFN-β-1b (8 MU sq qod). Early genes are IFN-regulatory factor-1 (IRF-1; enhances signaling) and IRF-2 (negative regulator), and later gene is 2′,5′-oligo-adenylate synthetase (2′,5′-OAS, an IFN-α/β-induced anti-viral enzyme). (Pre vs. Rx: IRF-1 and IRF-2, *p* < 0.02; 2′,5′-OAS, *p* < 0.03; paired *t*-test). (Avg ± SEM for IFN-stimulated gene/HPRT; RT-PCR) *N* = 5 RRMS patients for IRFs, and 10 for 2′,5′-OAS, vs. 9 normal (46).

**Figure 2:**
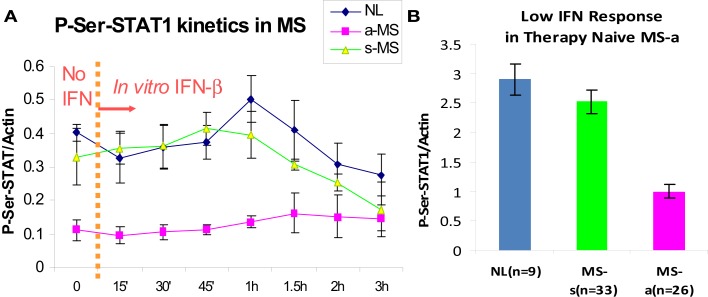
**(A)** P-S-STAT1 is markedly reduced in MNC from therapy-naïve, clinically active MS at baseline (left of dashed line), and after stimulation with 160 U/ml IFN-β (right of dashed line). **(B)** Area under curve of IFN-β-induced P-S-STAT1 is high in stable RRMS and healthy controls (NL) at 60′ vs. active MS (8 NL, 7 RRMS-s, 7 RR + SPMS-a) (*p* < 0.001 active/progressive MS vs. NL; ANOVA with repeated measures) (32).

The first patients that were placed on commercially available IFN-β showed induction of IFN-g (Th1), IL-10 (Th2) (46), and the IFN-stimulated proteins IRF-1, IRF-2, and 2′,5′-oligoadenylate synthetase (2′,5′-OAS) in mononuclear cells (MNCs) (49) (Figure 1). However, before any therapy, IFN regulation is abnormal in MS. Levels of these ISG products were actually low, and after IFN-β injections, levels rose only to levels seen in unstimulated control MNC. It was apparent that IFN responses were subnormal in MS, and that IFN-β therapy corrected IFN-induced protein levels back to the normal range. Extensive experiments using flow cytometry of IFNAR expression, Western blots of P-Y-STAT1, gel shift assays (EMSA), transfection of MNC with an IFN-responsive human ISRE reporter gene, and SHP levels to measure potential dephosphorylation of *P*-tyrosine, all showed that P-Y-STAT1 levels and P-STAT1 binding to DNA were normal and or above normal [Reder et al., 2000, unpublished and (32)] (Figure 2). Despite normal levels of P-Y-STAT1, however, induction of the above-mentioned ISG and MxA, an IFN-induced anti-influenza protein, was subnormal. It was later discovered there was a second STAT1 phosphorylation site at serine 727 (50). In unstimulated MNC from therapy-naive patients, P-S-STAT1 levels were low in MS (32). P-S-STAT1 was also poorly induced by IFN-β in cultured MNC, and this low P-S-STAT1 correlated with low levels of ISG. This indicated that there is a fundamental defect in IFN regulation that underlies MS, and that is likely to have consequences for immune regulation, therapy, and CNS repair.

P-S-STAT1 levels are low in MNC during exacerbations and progression (32). *In vitro*, induction of therapy-naïve MNC with IFN-β induces phosphorylation of STAT1 on serine in healthy controls and in patients with stable RRMS. In contrast, P-S-STAT1 is not induced during exacerbations and progression. A subset of downstream genes are not induced during the IFN-resistant state seen in active MS. MxA and viperin induction is diminished, but other ISG such as PKR have normal expression (32, 38).

The subdued IFN response that underlies clinical disease activity in therapy-naïve MS patients has consequences for immune regulation, and may also predict future disease activity. The IFN-resistant state appears to be corrected by IFN-β therapy. IFN-β injections increase IRF-1, IRF-2, 2′,5′-OAS, MxA, and viperin to near normal levels (Figures 1 and 2) (32, 46). Type I IFNs, themselves, are important IFN-stimulated proteins. IFN-β injections cause a rise in serum IFN-β that peaks at 30 min and then declines. This elevation is soon followed by a second, prolonged rise in serum type I IFN activity, presumably from newly induced IFN-α plus more IFN-β (38). Finally, IFN-β therapy of MS restores defective CD8 regulatory cell function (51), increases expression of the inhibitory ILT3 protein on monocytes (52), and reduces expression of costimulatory molecules on B cells (53).

Interferon-β-treated patients with ostensibly stable MS, yet weak responses to their IFN injections, are more likely to have attacks in the future (54). These weak responses parallel the effect of neutralizing antibodies (NAbs) to IFN-β, where high-titer serum NAb correlate with more MRI lesions during IFN therapy, presumably from lower circulating IFN-β levels. However, effects on clinical activity are difficult to demonstrate, likely due to complex pharmacokinetic effects of Abs to IFN (55).

Would more frequent or higher doses of IFN-β reverse the IFN signaling defect in those patients refractory to conventional doses of IFN-β? In a very large study, patients with stable RRMS did not have fewer relapses or MRI lesions from double doses of every-other-day IFN-β-1b. Thus, the approved (single) dose of IFN-β is optimal in early, stable RRMS. However, the higher dose was more effective in preventing black hole development (12). The IFN signaling defect is less common in stable RRMS than during active and progressing MS (12). With thrice-weekly IFN-β-1a, there is no difference in outcome between 22 and 44 μg doses in RRMS with low EDSS (early MS), but when the EDSS is >3.5 (later or more severe MS), the 44-μg dose is superior in preventing relapses and progression (56). This suggests complex interactions between IFN-β dose and injection frequency, NAb, and form or severity of MS.

Unexplored issues remain with effects of excessive or defective IFN signature and responses to therapy. Is the incidence of infections and cancer reduced by IFN therapy? In a large, but only 2-year, study, there were fewer bladder infections and cancers in the intramuscular IFN-β-1a subgroup compared to placebo and fingolimod (57). Does correction of low IFN levels restore immunity and promote neuroprotection? Is the aging process slowed with normal or high levels of IFN-β, a protein that is cell-protective?

## IFN Regulation in SLE, Sjögren’s Syndrome, and NMO is Opposite of Regulation in MS

Neuromyelitis optica is a demyelinating disease that until recently was simply considered a severe variant of MS. We have argued that NMO is actually much closer to SLE and Sjögren’s syndrome, and that it differs fundamentally from MS (Table 2) (47, 58).

CNS Sjögren’s syndrome affects younger women, 18–40 years-old, predominantly non-white, whereas the primary form affects 45–55-year-old women. The clinical appearance is similar to NMO (Devic’s disease), with devastating spinal cord or optic nerve lesions. Onset of devastating CNS symptoms is over hours to days, may follow infections, and recovery is often incomplete. Spinal cord lesions in CNS Sjögren’s syndrome and NMO span 3 or more vertebral segments, are central or holocord, are demyelinating but also necrotic, are sometimes so severe that there is spinal cord swelling. Optic nerve lesions are also highly destructive, and can have bilateral onset. In contrast, MS lesions are smaller, are <1 cord segment long, are often acentric or subpial, are not necrotic, and typically recover partially or completely, and optic neuritis is unilateral.

**Figure 3:**
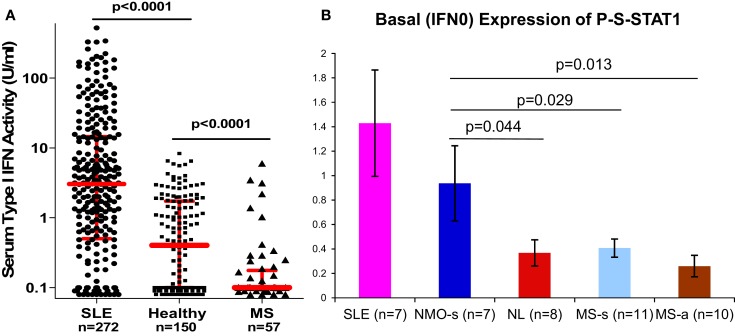
**(A)** Serum IFN-α/β activity is low in MS vs. NL, and high in SLE. IFN-α/β activity was obtained from real-time PCR of three expressed genes in WISH epithelial cells. *N* = 272 SLE on minimal Rx, 150 healthy controls, 57 stable RRMS naïve to Rx. Red bars = log median ± interquartile range (Kruskal–Wallis test). Medians = 3.1 for SLE, 0.4 for NL, and 0.1 for MS. Ninety percent of MS levels are <0.5 U/ml. In NMO, IFN-α/β is in the normal or SLE range. IFN-α/β detection is 0.1–0.5 U/ml, i.e., 20× more sensitive than many available assays (38). **(B)** P-S-STAT1 levels in MNC from SLE, stable NMO on minimal therapy, normal controls, stable RRMS, and active RRMS. Unpaired *t*-test with equal variance.

The type I IFN signature is supra-normal in NMO, but diminished in MS (38). NMO patients who were partially treated with low-dose steroids or plaquenil have normal levels of serum type I IFN activity (6× greater than in MS), and *in vitro* induction of P-Y-STAT-1, MxA, and viperin is excessive compared to healthy controls (Figure 3). In contrast, therapy-naïve MS patients have low levels of type I IFN activity and subnormal responses to IFN-β *in vitro*.

Neuromyelitis optica is now defined by MRI criteria – longitudinally extensive cord MRI lesions, and by serology – NMO-IgG positive. However, some cord lesions are too short on MRI and less devastating than expected, and only 60–70% of suspected NMO patients are NMO-IgG positive. Moreover, there is significant clinical overlap with CNS Sjögren’s disease, but here only 50% are NMO-IgG positive. Using serum type I IFN activity as a discriminator, ROC analysis shows a 35-fold ability to discriminate between NMO/CNS Sjögren’s and MS. This and other tests that could help discriminate between NMO and MS would aid in therapeutic decisions. This is important because disease mechanisms and responses to therapy differ between the two. For instance, IFN-β does not benefit or worsens NMO, but IFN-β is therapeutic in MS.

## IFN-β Therapy of MS: Cognitive Benefits, Prolonged Survival, Induction of Antioxidants and Possible Neuroprotection

One third of patients with pediatric-onset MS and clinically isolated demyelinating syndromes (CISs) are abnormal on neuropsychological testing. Untreated MS patients have significant cognitive loss over time, to the point of falling several *z*-scores below expected levels over 20 years (59). IFN-β improves cognition and slows cognitive loss in MS. The benefit was significant in the 2-year pivotal trials and later studies for all three forms of IFN-β-1. In the original IFN-β-1b trial, 32 patients tested over 2 years showed a dose-dependent improvement in visual-spatial performance compared to placebo (19). Benefit correlated with diminished MRI activity. The periventricular predilection of MS lesions could selectively disrupt the input and output of the calcarine cortex. We hypothesized that IFN-β quelled inflammation in these sites to enhance visuospatial performance (60).

Sixteen of these patients were studied in a long-term follow-up, 16 years after starting IFN-β (61). They did not remain in the original placebo, 50 μg, and 250 μg qod subgroups for more than 3 years, because at study end in 1993, all of these patients remained on IFN-β or started IFN-β *de novo* when the drug was approved. Fifteen of 16 patients were taking IFN-β at the 16-year point. The natural history of progressive decline of cognition in MS would lead one to expect loss of cognitive function after this long period, but there was no or only minimal cognitive decline at 16 years. This indicates that IFN-β-1b had a pronounced long-term benefit on cognition in active RRMS.

Interferon-β-1b therapy prevents death. In the pivotal IFN-β-1b study, there was a 5-year randomized disparity in placebo vs. IFN-β treatment, and then all patients started standard-of-care treatments. Five years of IFN-β therapy increased later survival by 7 years over a 21-year observation, a 47% reduction in mortality (62). What is the mechanism of the profound effect of IFN-β on survival in MS? Baseline pre-therapy male sex, high T2 MRI burden of disease, and high EDSS all were increased in those who died, but IFN-β-1b had an independent effect (nearly 50% reduction in mortality) for each variable. On-study responses such as new relapses, T2 lesions, or neutralizing Ab titers, did not significantly change mortality. This suggests that IFN-β has effects on MS that are beyond the usual trial readouts of relapses and MRI lesions.

Over the first 16 years of this study, those who were on IFN-β-1b therapy for >80% of the time (restricted to a subset of the original 250 μg IFN-β group), compared to those treated only <10% of the time (a subset of the original placebo) were ∼60% less likely to require a cane or wheelchair or to develop SPMS (63). The delay of onset of SPMS in the low medication usage group was 12 year, vs. 18 year in the high medication usage group. Note that these data are based on correlations, and other factors could influence medication use.

Interferon-β’s benefit on mortality in MS cannot be explained by a simple Th1–Th2 shift. Could IFN-β induce genes that would protect or repair brain cells? The likelihood that type I IFNs evolved to have cytoprotective and neuroprotective effects was introduced in the Prolog. Can we measure gene induction after IFN-β therapy?

Multiple sclerosis patients with carefully defined clinical disease activity who had been on prolonged IFN-β-1b therapy were studied more than 60 h after their last injections, and then exactly 4, 18, and 42 h after an injection of IFN-β. RNA from MNC was run on Affymetrix Hu133A and also all-exon arrays. IFN caused a rapid (4 h) and intermediate (18 h) induction of ISGs at 4 and 18 h, and levels then fell back to baseline at 42 h (33). Surprisingly, after the 60-h washout, there remained an RNA signature for upregulation of scores of genes that control fatty acid oxidation, apoptosis, energy metabolism, and cytoprotection, such as Nrf2 (64). These patients had been on IFN-β-1b therapy for 2–18 years. It is likely that long-term IFN-β therapy alters the set-point for control of cell metabolism and neuroprotection in blood and CNS cells. Glucocorticosteroids, stress, smoking, and obesity increase oxidative stress. Some of these environmental factors increase MS severity. Perhaps induction of cytoprotective genes explains some of the benefits of IFN-β-1b therapy on MRI black hole formation, cognition, and survival.

Multiple sclerosis brain lesions are an ominous predictor of shorter survival (62). MRI lesions predict mortality in untreated and treated MS patents. IFN-β therapy reduces new MRI T2 and new Gd+ lesions by 85% (48, 65) and reduces formation of permanent T1 MRI lesions (“black holes”) (12). Black holes are a marker for inflammatory damage as well as lack of repair. IFN-β therapy prevents inflammation and may enhance repair. IFN-β also increases NAA concentration in brain neurons on MRS imaging (66), presumably by rescuing unhealthy neurons from death. IFN-β-1b therapy in MS has delayed benefits on cognition (19). Though speculative, fewer MRI lesions during therapy may increase survival. CNS lesions could disrupt trophic outputs to the rest of the body or disrupt immune regulation. For instance, sympathetic nerves bathe the spleen and secondary lymphoid organs with inhibitory catecholamines and neuropeptides. Spinal cord lesions that disrupt these SNS pathways will disinhibit the immune system, provoke and amplify autoimmune disease, and correlate with presence of progressive forms of MS (67, 68). Thus, new MRI lesions, permanent T1 black holes, cold purple feet from SNS damage in SPMS, cognitive loss, and even death may be interrelated phenomena and appear to be thwarted by IFN-β therapy.

## Combination Therapy with Other Agents Can Perturb the IFN Signaling Pathway

Intrinsic or disease-specific responses to IFN can modify the course of an autoimmune disease, but can be altered by exogenous triggers or by IFN-β therapy. Viruses for instance, cause exacerbations of MS, likely by activating not just IFNs, but many other facets of immunity. Could other agents modify intrinsic IFN levels or the effects of therapy? Two examples include statins and vitamin D.

**Figure 4:**
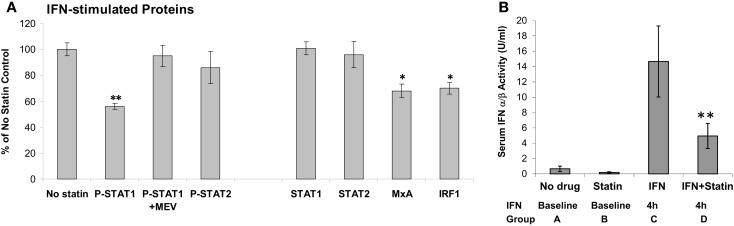
**(A)** *In vitro* atorvastatin reduces IFN-β effects. MNC from 21 therapy-naïve RRMS were pretreated 24 h with 10 μM atorvastatin, then induced with 160 U/ml IFN-β-1b for 45 min (phosphorylated/activated P-STAT transcription factors) and for 24 h (induced unphosphorylated STAT1, STAT2, also MxA and IRF-1). Proteins quantified with Western blots, normalized with actin. Percentage change = statin-treated vs. no-statin (100%). *p < 0.05, **p < 0.001 vs. no-statin control. MEV = 100 μM mevalonate to reverse statin effect. Mean ± SEM; 21 replications (38). **(B)** *In vivo* statins reduce IFN-β therapy induction of serum type I IFN activity. Sera were obtained at 8 a.m. after statin washout or long-term statin alone, and then exactly 4 h after IFN-β injections or high-dose statins plus 4 h IFN-β. Fourteen stable RRMS. **p < 0.001 vs. IFN alone (paired *t*-test). Mean ± SEM (38).

Statins lower cholesterol, but are also anti-inflammatory. It would seem reasonable to combine statins with IFN-β to treat the inflammation of MS. Statins appeared to reduce MRI lesions (69). However, only active patients entered this uncontrolled study, and the 41% reduction in MRI activity is what would be expected due to regression to the mean without any therapy (70). Several small studies seemed to show benefit of combining statins and IFN-β. However, a controlled trial of RRMS patients on subcutaneous IFN-β-1a therapy who were then randomized to IFN-β alone or IFN-β plus high-dose atorvastatin (40–80 mg/day) showed that the combination provoked MRI and clinical exacerbations (33). A larger study of IFN-β-1a ± atorvastatin showed similar trends, especially in a composite score of MRI and clinical MS activity (71). The presumed synergy between two anti-inflammatory agents did not materialize because statins actually block IFN signaling. *In vitro*, statins cause a dose- and time-dependent block of IFN-β-induced phosphorylation of STAT1 (38) (Figure 4). *In vivo*, patients on various IFN-β therapies plus various forms of statin were tested after washing out both drugs and then performing IFN-β induction kinetics after: (1) adding IFN-β alone, and later repeating with (2) IFN-β plus high-dose statins. A dose of statins blocked IFN-β formation of P-Y-STAT, but not P-S-STAT1, and inhibited production of downstream proteins such as MxA and viperin. This cautionary tale suggests that statins could block the benefit of IFN-β, and that if both are needed, then the posology of therapy should be adjusted so statins are given at a time when they will have the least effect on the IFN injection.

Vitamin D has pleiotropic effects on immunity. It enhances macrophage function, but also induces IL-10. Serum vitamin D levels vary, due to lifestyle, skin color, and seasonal sunlight. Low levels are linked to onset of MS, and exacerbations once MS has developed. Could this inexpensive, sometimes free, agent be added to IFN-β? Several small studies show no additive effects, including one that was designated as class I evidence by the journal, Neurology (72). Unfortunately, a significant mismatch in the baseline demographics favored IFN-β monotherapy and the results are not at all conclusive. Another study showed no additive effect with subcutaneous IFN-β-1a (73). In a larger controlled study, however, vitamin D3 added to IFN-β-1b reduced new MRI lesions compared to IFN-β alone (74). IFN-β also appears to increase serum vitamin D levels (75). Many other studies of vitamin D plus IFN-β in MS, and vitamin D alone in SLE, are in progress. It is hoped that current, partially effective and expensive, therapies will be enhanced by a second inexpensive agent.

## Summary

- Type I Interferons (IFN-α/β) control viruses, cancer, cell proliferation, and immunity
- Type I IFNs were present in early fish, 400 million years ago
- Interferons evolved complex responses to viruses over eons; vertebrate survival likely was enhanced by cytoprotective effects of IFNs
- Interferons regulate ∼1000 genes
- Plasmacytoid dendritic cells produce IFN-α and some IFN-β; fibroblasts produce IFN-β
- The IFNAR activates the STAT1 transcription factor on tyrosine, allowing DNA binding
- Some genes are further induced by a second phosphorylation on serine (P-S-STAT1)
- Viruses bind TLR, RIG-I, and MDA5. These activate IRF3, then IFN-β and IFN-α1, then IRF7, then many subtypes of IFN-α
- Viruses, exogenous, and perhaps endogenous, trigger IFN production
- High serum and organ IFN levels are linked to SLE, Sjögren’s syndrome, Aicardi–Goutières, and Cree encephalitis, as well as CNS Sjögren’s and NMO
- Interferon-α treatment triggers and amplifies SLE
- Serum IFN levels are low in MS
- Responses to *in vitro* IFN-β are low in MNCs from untreated MS patients during clinical activity and progression
- Responses to *in vivo* IFN-β therapy are low in MS during clinical activity and progression, measured by gene induction and activation of transcription factors
- Low responses to IFN during clinically active MS are linked to low levels of P-S-STAT1
- Interferon-β is therapeutic in MS, and may be most effective in patients with low IFN responses
- Some drugs such as statins, block type I IFN signaling, and worsen MS
- The high IFN responses in NMO and CNS Sjögren’s disease can be used to differentiate them from the low responses in MS

## Conflict of Interest Statement

Bayer Schering Pharma, Biogen, Ezose, Genzyme, Genentech, MedImmune, Novartis, Questcor, Sanofi-Aventis, Serono, Teva – Travel expenses and time are covered to attend advisory boards and scientific meetings; Grant support; Clinical trials; and Data safety and monitoring boards for clinical trials and development of therapies for MS. NMSS – Travel expenses for grant reviews.
